# *FGF14* GAA Intronic Expansion in Unsolved Adult‐Onset Ataxia in the Care4Rare Canada Consortium

**DOI:** 10.1002/acn3.70016

**Published:** 2025-04-07

**Authors:** Alexanne Cuillerier, Giulia F. Del Gobbo, Layla Mackay, Erika Wall, Madeline Couse, Laura M. McDonell, Mireille Cloutier, Matt C. Danzi, Jodi Warman‐Chardon, Pierre R. Bourque, Oksana Suchowersky, Alan Mears, Luke Seldenthuis, Wendy Mears, Laura Larrigan, Alexandre White‐Brown, Gerald Pfeffer, Dennis E. Bulman, David Dyment, Care4Rare Canada Consortium, Kym M. Boycott

**Affiliations:** ^1^ Children's Hospital of Eastern Ontario Research Institute Ottawa Ontario Canada; ^2^ University of Ottawa Ottawa Ontario Canada; ^3^ Centre for Computational Medicine, the Hospital for Sick Children University of Toronto Toronto Ontario Canada; ^4^ Department of Genetics Children's Hospital of Eastern Ontario Ottawa Ontario Canada; ^5^ Dr. John T. Macdonald Foundation Department of Human Genetics and John P. Hussman Institute for Human Genomics University of Miami Miller School of Medicine Miami Florida USA; ^6^ Department of Medicine, Neurology The Ottawa Hospital Ottawa Ontario Canada; ^7^ Ottawa Hospital Research Institute Ottawa Ontario Canada; ^8^ Faculty of Medicine, Department of Neurology, Pediatrics, Psychiatry and Medical University of Alberta Edmonton Alberta Canada; ^9^ Newborn Screening Ontario Ottawa Ontario Canada; ^10^ Alberta Child and Health Research Institute, Department of Medical Genetics, Cumming School of Medicine University of Calgary Calgary Alberta Canada; ^11^ Hotchkiss Brain Institute, Department of Clinical Neurosciences, Cumming School of Medicine University of Calgary Calgary Alberta Canada; ^12^ Department of Medical Genetics University of Alberta Edmonton Alberta Canada

## Abstract

**Background and Objectives:**

Spinocerebellar ataxias (SCA) represent a clinically and genetically heterogeneous group of progressive neurodegenerative diseases with prominent cerebellar atrophy. Recently, a novel pathogenic repeat expansion in intron 1 of *FGF14* was identified, causing adult‐onset SCA (SCA27B). We aimed to determine the proportion of our unsolved adult‐onset ataxia cohort harboring this expansion using several technologies, and to characterize the phenotypic presentation within our population.

**Methods:**

Individuals presenting with adult‐onset ataxia (> 30 years old) and negative previous genetic testing were selected from the Care4Rare patient repository. Affected individuals were from all ethnicities, and 90% had a family history suggestive of dominant ataxia, representing 19 of the 23 families included. We used multiple tools (PCR, long‐read genome sequencing and optical genome mapping (OGM)) to identify the pathogenic GAA repeat in *FGF14*.

**Results:**

Of the 23 families included in this study, 65.2% harbored a pathogenic GAA expansion in *FGF14*. Individuals of French‐Canadian descent (FC) represented most of our cohort and had a 64.7% diagnostic yield. Affected individuals presented with gaze‐evoked nystagmus, gait ataxia, cerebellar dysarthria, and early episodic features. The GAA expansion in *FGF14* was visible by OGM in all individuals tested.

**Interpretation:**

Our diagnostic yield demonstrates this expansion may be the most common cause of adult‐onset SCA in dominant families of FC ancestry. Our FC participants have a phenotype distinct from previously published FC patients, with gaze‐evoked nystagmus being the most common eye anomaly. From a diagnostic standpoint, the pathogenic GAA repeat can be identified by OGM, but additional tests are required to complement the interpretation.

## 1 Introduction

Spinocerebellar ataxias (SCA) form a group of progressive neurodegenerative diseases that are highly heterogeneous both clinically and genetically. Patients present with progressive atrophy of the cerebellum and brainstem primarily leading to unsteady gait, clumsiness, and dysarthria, but also with variable additional features delineating ataxias into over 35 subtypes. Altogether, SCAs have an estimated prevalence of 1 in 33,000 worldwide, and approximately 75% of these patients are without a molecular diagnosis and are considered “unsolved” [1, 2, 3]. The advancement of both bioinformatics tools and long‐read genome sequencing (LR‐GS) technology led to the recent identification of a pathogenic GAA repeat expansion in intron 1 of *FGF14* in patients with autosomal dominant spinocerebellar ataxia type 27B (SCA27B) [4, 5]. Since then, several groups have screened cohorts of unsolved patients presenting with adult‐onset ataxia and cerebellar atrophy and identified this pathogenic tri‐nucleotide expansion in individuals of different ethnic backgrounds, suggesting it arose on multiple different haplotypes [4, 5, 6, 7, 8, 9, 10, 11, 12, 13]. Here, we investigated the contribution of the newly discovered pathogenic GAA repeat expansion in intron 1 of *FGF14* in a cohort of patients presenting with unsolved adult‐onset ataxia from the Care4Rare Canada research program. We used a combination of various tools, including long‐range PCR (LR‐PCR), LR‐GS, and, for the first time, optical genome mapping (OGM), to assess the presence of a pathogenic expansion in intron 1 of *FGF14*.

## 2 Methods

### 2.1 Ethics, Enrollment and Clinical Assessment

The Care4Rare Canada Consortium is a national collaborative research effort to discover new mechanisms to explain unsolved rare genetic disease [14]. All participants in this study were enrolled in the research program after standard‐of‐care genetic testing had failed to identify a molecular etiology. This study was approved by the institutional research ethics board (Children's Hospital of Eastern Ontario; #11/04E and CT‐O1577) and informed consent was obtained from all participants at enrollment. Patients with a progressive adult‐onset ataxia after the age of 30 years, with either a positive family history that suggested autosomal dominant (19 families, 83%) or with no family history available (4 families, 17%), were included in this study. Participants of all ethnicities were included, and their ancestry was self‐reported. Patients with a positive result were re‐evaluated, when possible, to systematically characterize their clinical features and to proceed to confirmatory clinical testing.

### 2.2 Molecular Testing

The intronic expansion in *FGF14* was investigated in the Care4Rare research laboratory by LR‐PCR, using genomic DNA and primers flanking the known pathogenic locus in intron 1 (F: TGCAAATGAAGGAAAACTCTT, R: CAATGATGAATTAAGCAGTTCC) [4]. The Phusion Hot Start II High Fidelity polymerase kit (Thermo Fisher) was used to amplify the region of interest and the product was run by electrophoresis at 40 V for 3–4 h on a 1% agarose gel. Post‐run gels were incubated in Gel‐Red solution (5%) for 20 min before imaging (ChemiDoc, BioRad). Bands larger than 750 bp, corresponding to 250 GAA repeats, were considered pathogenic. Each genomic DNA sample was tested twice in separate experiments. Clinical confirmatory testing was ordered by physicians and performed by the University of Chicago Genetic Services Laboratory; sizing was performed by flanking‐PCR and capillary electrophoresis to amplify across the repeat region in *FGF14*, followed by repeat‐primed PCR (RP‐PCR) and capillary electrophoresis to amplify within the repeat region in *FGF14*.

### 2.3 Long‐Read Genome Sequencing

PacBio HiFi LR‐GS was performed on high‐molecular weight DNA extracted from blood obtained from seven individuals from two families in this cohort to a genome‐wide coverage of a minimum 30×. Library preparation, sequencing, and read alignment methods have been described in detail elsewhere [15]. Genotyping and sizing of alleles within the *FGF14* GAA repeat locus was performed using Tandem repeat genotyping and visualization (TRGT) v0.7.0 in all individuals, and the region was further characterized through visual inspection of aligned HiFi reads in BAM files in the Integrative Genomics Viewer (IGV). In a secondary analysis, we re‐processed raw sub‐read data by adjusting the default parameters for circular consensus sequencing to emit a consensus sequence for every zero‐mode waveguide (ZMW), even when it fell below standard quality thresholds (using the flag—all) and then re‐analyzed the *FGF14* GAA repeat locus using TRGT with the—min‐read‐quality parameter set to ‘−1’ followed by manual inspection of BAM files.

### 2.4 Optical Genome Mapping

OGM was performed on ultra‐high molecular weight (UHMW) genomic DNA extracted from blood following the manufacturer's protocol (SP‐G2, Bionano Genomics, San Diego, USA). Labeled and stained UHMW genomic DNA was loaded on chips, and linearization and imaging of molecules were performed on the Saphyr (Bionano Genomics, San Diego, USA). For each sample, 1000GB of data was collected and processed by the *de novo* pipeline. Intronic expansions in *FGF14* were reported as insertions (structural variants), corresponding to the difference between two labels on the consensus maps compared to the reference (GRCh38/hg38).

### 2.5 Statistical Analysis

Figures were generated and statistical analyses were performed using GraphPad Prism. The correlation between age at onset and repeat size was determined by the calculation of the Pearson correlation coefficient.

## 3 Results

### 3.1 Cohort Demographics and Previous Genetic Testing

**FIGURE 1:**
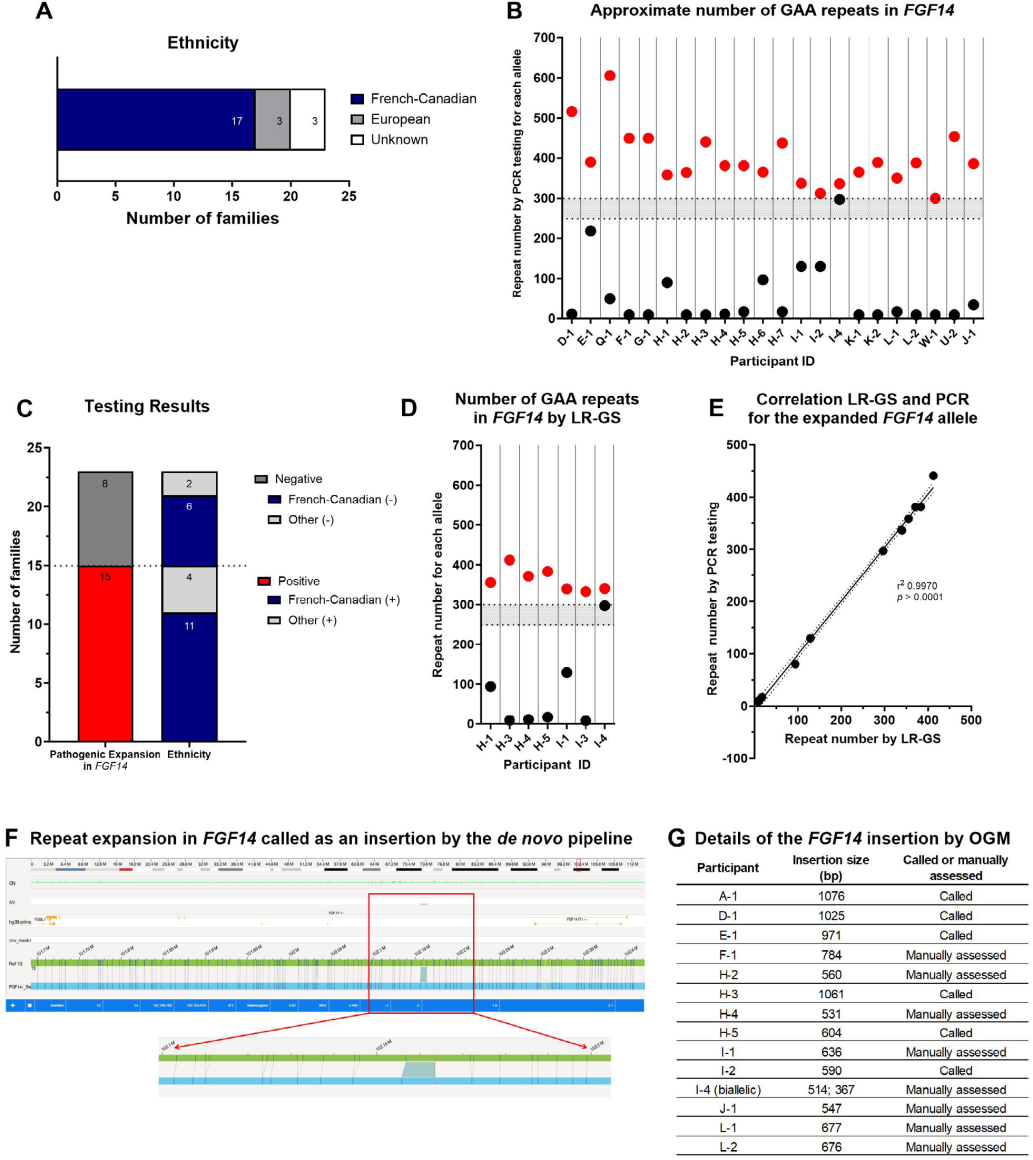
Molecular characterization of the Care4Rare Canada unsolved adult‐onset ataxia cohort. (A) Self‐reported ethnicity of the families included in our Care4Rare unsolved dominant adult‐onset ataxia cohort. (B) Approximate allele size obtained by flanking PCR (clinical testing) in 22 participants. Red dots represent allele ‘a’ (pathogenically expanded, > 300 repeats) and black dots represent allele ‘b’. The gray zone represents the incomplete penetrance range of 250–299 repeats. (C) Overall results of molecular testing for the pathogenic expansion in FGF14, where red represents the positive diagnosis and dark gray the negative ones. The proportion of FC families, in blue, is depicted for both positive and negative results. (D) Approximate allele sizes obtained from the tandem repeat genotyping tool TRGT in seven participants tested by HiFi LR‐GS. (E) Correlation of repeat number, between LR‐GS and RP‐PCR. (F) Representative screenshot of the FGF14 gene in the genome view in Bionano Access. The region in blue represents the insertion called by the de novo pipeline. (G) Size of the insertion in FGF14, corresponding to the repeat expansion and if the insertion was either called by the *de novo* pipeline or assessed manually, for each participant tested by OGM.

We selected individuals from the unsolved cohort of the Care4Rare Canada research program with adult‐onset cerebellar ataxia (> 30 years of age) and either a positive family history suggestive of autosomal dominant inheritance or a family history that was unavailable. In total, 42 affected individuals from 23 families were identified. Previous genetic testing included standard‐of‐care clinical testing for all affected individuals (common repeat expansions (SCA 1, 2, 3, 6, 7, 8, 17), ataxia panels and/or clinical exome sequencing) and additional research analysis of exome data or short‐read genome sequencing (SR‐GS) for some participants, all of which did not identify a pathogenic variant. More than half of our cohort is of self‐identified FC origin (17 families, 34 participants); one participant is of mixed FC origin; three families (four participants) are European; and three families (five participants) for which the ethnicity was unknown, or the information was not collected (Figure 1A).

### 3.2 Molecular Analysis of the FGF14 GAA Repeat Expansion by Multiple Approaches

#### 3.2.1 PCR Analysis

We used a combination of LR‐PCR utilizing primers flanking the region of interest in the research lab, and flanking‐PCR combined with RP‐PCR through clinical testing, to assess the contribution of the intronic expansion in intron 1 of *FGF14* in our cohort of participants with unsolved ataxia. Of the 23 families, research and/or clinical testing identified 15 families (31 individuals) with the *FGF14* intronic expansion for a diagnostic yield of 65.2%. Accurate repeat size was available in eight of these families (22 participants) (Figure 1B). The average expansion size is approximately 398 repeats, and one participant carries biallelic expansions, comprising one allele with 336 repeats and the other with 297 repeats. Another participant carries a pathogenic allele with 390 repeats and another allele with 218 repeats, which falls in the sub‐pathogenic range. The largest pathogenic expansion identified in this study was 606 and the smallest 297 repeats. Eleven of the 15 positive families were of FC origin, for a diagnostic yield of 64.7% in this specific population (Figure 1C).

#### 3.2.2 Long‐Read Genome Sequencing

We performed PacBio HiFi LR‐GS in seven individuals from two families in this cohort (Family 407B and 407 K). Initial application of the tandem repeat genotyping tool (TRGT) to the *FGF14* repeat locus led to the detection of pathogenic expanded alleles in four of seven individuals. Upon manual inspection of BAM files in IGV, we noted poor coverage of these expanded alleles, and the phasing of reads and coverage in the region in the three individuals without detectable expansions suggested that dropout of expanded GAA alleles was likely (Figure S1). To address the possibility of allele dropout of long low‐complexity GA‐rich sequences in HiFi data, we re‐analyzed the raw subread data, loosening filters for circular consensus sequencing to recover any lower‐quality HiFi reads containing the expanded GAA sequences. This successfully recovered expanded alleles in all seven individuals. We applied TRGT to this revised HiFi data to genotype the *FGF14* repeat locus and confirmed each affected individual carried at least one pathogenic fully penetrant allele of > 300 repeats, all of which were pure (GAA)_n_ repeats (Figure 1D). In one affected participant, we confirmed compound heterozygosity of the *FGF14* expansion as was seen in PCR analyses, with TRGT estimating allele sizes of 297 and 340 GAA repeats compared to 297 and 336 reported by the clinical laboratory using flanking‐PCR combined with RP‐PCR. In six of seven participants, allele sizes determined by clinical testing were available and were highly concordant with those determined by LR‐GS (Pearson *R* = 0.9985; Figure 1E), confirming the ability of LR‐GS to accurately size and determine the sequence composition of the *FGF14* GAA repeat.

#### 3.2.3 Optical Genome Mapping

OGM can detect repeat expansions if the repeat size is above the detection threshold of 500 bp. The smallest pathogenic intronic repeat expansion in *FGF14* is 250 triplets, corresponding to 750 bp, which is above the limit of detection. To test the potential of OGM to detect the intronic GAA repeat expansion in *FGF14*, we collected blood samples from 14 participants who had a positive clinical genetic testing result within our cohort. The intronic expansion in *FGF14* was called by Bionano's *de novo* pipeline as an insertion (Figure 1F) in six of the 14 participants. Upon manual inspection of the data for the remaining eight participants, the expansion was visible in the consensus maps, and we were able to determine the size of the insertion in the first intron of *FGF14* by calculating the distance between two labels flanking this region and comparing this value to the reference. Overall, OGM allowed the visualization of insertions corresponding to the pathogenic expansion in *FGF14*, ranging from 367 to 1076 bp in our participants (Figure 1F). The participant with the biallelic expansions in *FGF14* was also tested by OGM, and the expanded alleles correspond to insertions of 514 and 367 bp each based on label position and distance in between (Figure 1G).

### 3.3 Haplotype Analysis

Given the high proportion of affected FC participants in our cohort, we set out to investigate whether there was a common haplotype associated with the *FGF14* expansion shared among our FC participants. Using the haplotype‐phased LR‐GS data from the two families sequenced, we confirmed that they shared at minimum a 688 Kb‐long haplotype block that encompassed *FGF14*, which included 565 shared variants. Additionally, extrapolating this to the additional two FC families with SR‐GS data available that were positive for expansions in *FGF14* and two FC families with SR‐GS data that were negative, we confirmed that this expansion‐associated haplotype was present in the two families with the expansion and absent from the two without. We then compared this haplotype with a candidate haplotype identified in FC patients from Quebec by Pellerin et al. 2023 [4] (Table S1) and it was only partially shared, suggesting the possibility of two expansion‐associated haplotypes in the FC population or, alternatively, that a shared haplotype has undergone significant divergence over time.

### 3.4 SCA27B Phenotype in the Care4Rare Cohort

**FIGURE 2:**
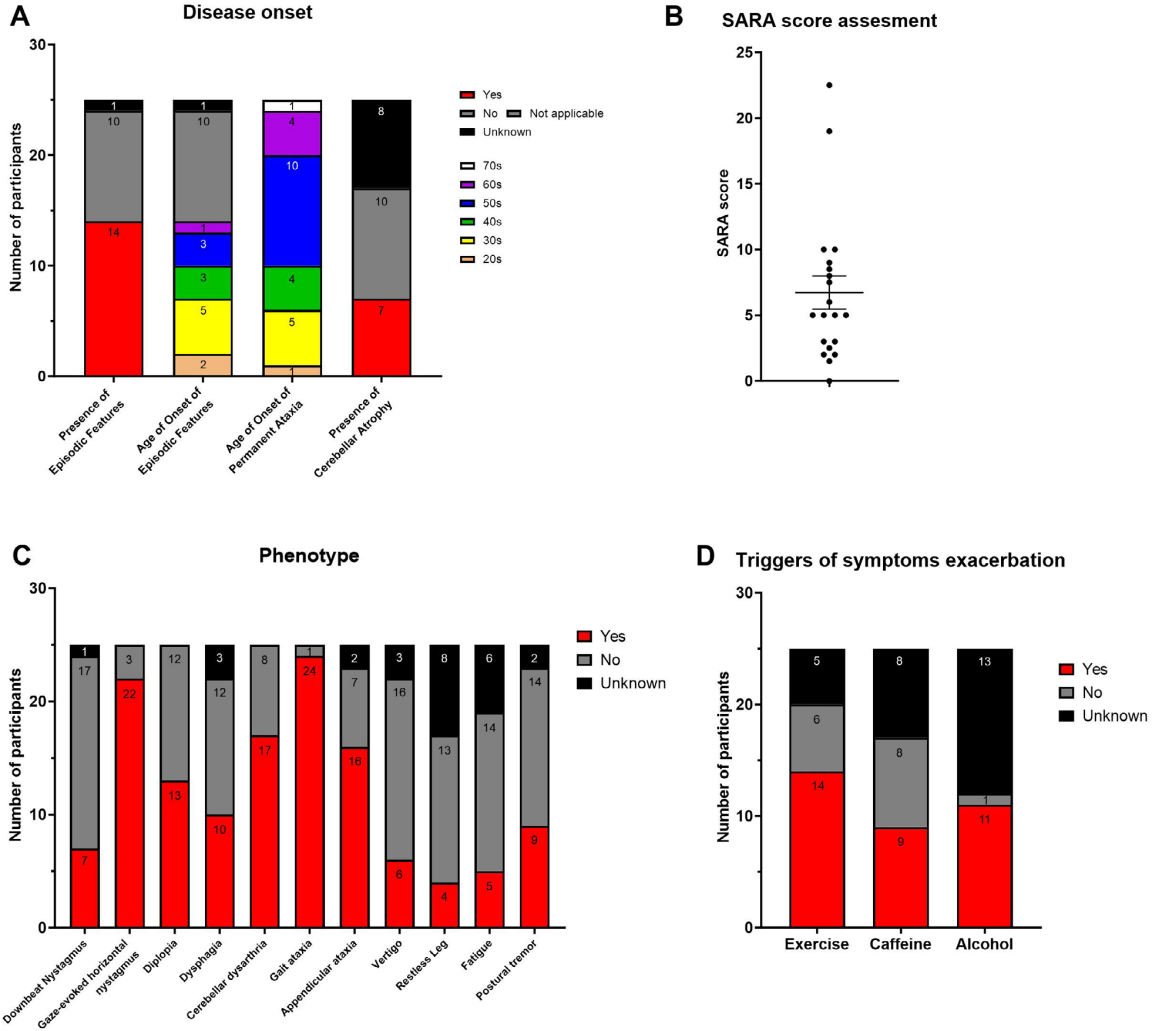
Clinical characterization of the SCA27B cohort. (A) Distribution of the presence of episodic features (EF), the age at onset of EF, the definitive age at onset of ataxia and presence of cerebellar atrophy by MRI in the 24 deeply‐phenotyped participants. Red represents yes (feature present), dark gray represents no (feature absent) and black represents unknown (either unrecognized by participant or MRI not performed). Decades of life are represented by orange (second decade), yellow (third decade), green (fourth decade), blue (fifth decade), purple (sixth decade), and white (seventh decade). (B) Distribution of SARA score in our deeply‐phenotyped participants. Each dot represents a value (corresponding to 1 participant), the line represents the mean, and the error bars represent the standard error of the mean (SEM). (C) Detailed phenotype and (D) known ataxia triggers assessed in our deeply‐phenotyped cohort. Red represents yes (feature present), dark gray represents no (feature absent) and black represents unknown (either not recognized by participant or, for the triggers, does not exercise, does not drink caffeinated beverages or alcohol).

We clinically characterized 25 participants (from 12 families) who were positive for the pathogenic intronic expansion in *FGF14* (Table S2). Of these 25 participants, 21 self‐reported as FC. Disease onset was in the 5th decade in 40% (10/25 participants). Hallmark features of SCA27B include episodic features as the first disease presentation in 56% of participants (14/25), manifesting for the first time in their 3rd decade for 36% of them (5/25). Cerebellar atrophy by MRI was reported in 41% (7/17) of participants who had imaging (Figure 2A). The Scale for Assessment and Rating of Ataxia (SARA) score was assessed in 20 participants, with an average score of 6.7 (Figure 2B). Downbeat nystagmus, gaze‐evoked horizontal nystagmus, and diplopia were present in 28% (7/25), 88% (22/25), and 52% (13/25) of assessed participants, respectively. Dysphagia was reported in 40% (10/25) and cerebellar dysarthria in 68% (17/25). Gait ataxia and appendicular ataxia were present in 96% (24/25) and 64% (16/25) of participants, respectively. Finally, vertigo, restless leg syndrome, fatigue, and postural tremor were reported in 24% (6/25), 16% (4/25), 20% (5/25), and 36% (9/25), respectively (Figure 2C). Participants reported their symptoms were exacerbated by exercise in 70% (14/20 who reported exercising), caffeine in 53% (9/17 who reported consuming caffeine), or alcohol in 92% (11/12 who reported consuming alcohol) (Figure 2D). We investigated if there was a relationship between age at onset and the repeat number of the *FGF14* pathogenic allele in a subset of 19 participants from our cohort for whom we had accurate allele size and found no significant correlation between these two factors (Pearson *r* = 0.390, *p* = 0.098).

## 4 Discussion

We performed a comprehensive assessment of 23 families (42 participants) from our Care4Rare unsolved adult‐onset ataxia cohort for pathogenic GAA repeat expansions in the first intron of *FGF14* using multiple tools, including for the first time, OGM. We identified pathogenic expansions in 15 families (31 participants), for a positive diagnostic yield of 65.2% in our cohort that is enriched for individuals with familial recurrence and for those of self‐reported FC ancestry.

Our cohort phenotype contributes to the emerging clinical understanding of SCA27B, with disease onset in the fifth decade for the majority of our participants [4, 5, 6, 7, 8, 9, 11, 12]. The FC subset of our cohort differed from the previously reported FC cohort from Quebec [4, 13], with approximately half the prevalence of downbeat nystagmus (30% vs. 66%) and a higher prevalence of gaze‐evoked nystagmus (86% vs. 57%). All three known triggers, exercise, caffeine, and alcohol, were reported to exacerbate symptoms in our participants. Of these, alcohol was the most common trigger among our participants, reported as a trigger in all but one participant who consumed alcohol. Several participants with milder symptoms reported exercising regularly. A number of studies have shown exercise can lead to neuronal adjustments through various mechanisms, leading to improved motor performance and gait in participants with chronic neurological conditions [16, 17]. Future studies detailing an optimized exercise program and quantifying the impact on ataxia symptoms would be relevant and could be beneficial to patients with adult‐onset slowly progressive ataxia.

We did not observe a correlation between the age‐at‐onset of ataxia and the number of repeats in *FGF14*, consistent with other studies reported to date [4, 9, 11, 18]. In fact, our participant with the largest expansion (606 repeats) had an onset of ataxia in their 60s, among the oldest at disease‐onset in our cohort. Inversely, our participant with the earliest presentation, in their 20s, had 390 repeats in *FGF14*, which is below the average repeat number of 398 found in our cohort. Therefore, our findings support the hypothesis of the presence of other factors, still unknown, impacting the age‐at‐onset of SCA27B.

Two affected individuals in our cohort suggest a correlation between biallelic expansions, age of onset, and severity. The age at onset of ataxia for our biallelic participants was in their 40s, among the youngest in our cohort, and their SARA scores were 19 and 8, above the average score of 6.5 in our cohort. A younger age at onset and a potentially more severe phenotype have been previously reported in two pairs of siblings carrying biallelic expansions [19]. The expansions were greater than 500 repeats in both sets of siblings, and they presented in their second decade. Based on these observations and our results, biallelic expansions in *FGF14* are likely associated with an earlier presentation and a more severe phenotype. Additionally, all affected individuals in our cohort had 300 or more repeats in *FGF14*, supporting 300 as a pathogenic threshold [20].

Investigation of the unsolved Care4Rare cohort revealed a positive diagnostic yield of 65.2%, and 64.7% in our FC families. Interestingly, the positive diagnostic yield varies greatly across the published reports, ranging from 1.2% in a Japanese cohort [12] to 61% in a previous FC cohort [4]. The studies with the lowest diagnostic yield (1.2% [12], 9% [9], 12% [6], 12.7% [18], 13.7% [5], 14.4% [5], 17% [7], 21.6% [11]) have either investigated only sporadic cases or have over 50% sporadic cases in their cohort. Similarly, studies with a high diagnostic yield (51% [10], 61% [4]) have a greater proportion of familial cases. The cohort here was heavily biased for familial cases. Consequently, our positive diagnostic yield is in the upper range and compares to other studies with a high proportion of familial cases [4, 10]. Moreover, our positive diagnostic yield of 64.7% in our FC families is in line with this variant being the most common genetic cause of adult‐onset ataxia in this population [4, 21].

The pathogenic intronic expansion in FGF14 has been shown to arise on multiple haplotype backgrounds, affecting individuals of European and Asian ancestry across the globe [4, 5, 6, 7, 8, 9, 10, 11, 12, 13]. Because of the high proportion of FC patients with a pathogenic expansion in *FGF14*, it has been suggested that this is secondary to a founder effect in this population [4]. In four FC families with available short‐read or long‐read genome sequencing data, we confirmed a shared expansion‐associated haplotype which was absent from two FC families who did not carry an expansion. This haplotype is not completely shared with that described in FC patients from Quebec by Pellerin et al. [13, 22]. This suggests that our FC population, which is mainly from northern Ontario and the Ottawa Valley, may have a different founder or, more likely, has diverged from the original founder in Quebec many generations ago.

The present study includes a family (Family 407 L; Table S2) previously reported to carry a potential disease‐causing variant in *PUM1* (Family X; NM_001020658.2: c.3103A>T (p.Thr1035Ser)) associated with the adult‐onset ataxia segregating in a multi‐generational family [23]. The authors at the time hypothesized the mode of inheritance of this *PUM1* variant was autosomal dominant with incomplete penetrance in this family. However, we have identified a pathogenic GAA expansion in *FGF14* in all affected family members, and it is absent in the presumably nonpenetrant unaffected family member initially reported. Moreover, this family's FC ancestry and phenotypic presentation are consistent with SCA27B, ruling out that the missense variant in PUM1 is causative and indicating that variants in *PUM1* are likely not associated with adult‐onset ataxia.

Only pure GAA repeats in *FGF14* have thus far been observed in affected individuals with SCA27B, and large expansions of different motifs at this repeat have been observed in controls; therefore, it is expected that only pure GAA expansions are pathogenic [4, 5, 6, 7, 8, 9, 10, 11, 12]. Therefore, LR‐GS is the most reliable approach to identify repeat expansions in *FGF14*, and indeed in this study we found that following a modified bioinformatic analysis, we were able to detect expanded alleles in all seven participants assessed by HiFi LR‐GS (PacBio) and accurately assess the size and sequence composition of the repeat. A description of the optimal testing for the detection of GAA repeat in *FGF14* was published in the last year and provides details of the best strategy available when LR‐GS is not easily accessible [7].

We tested the potential of OGM to detect the pathogenic repeat expansion in *FGF14*. The insertion was visible in all tested samples, but OGM did not call the insertion in eight of the 14 samples, for which manual inspection was required to obtain the insertion size. The results obtained by OGM do not correspond to the size of the entire GAA‐repeat sequence in *FGF14*, but to the difference between the sample and the reference sequence, without information of the repeat size in the Bionano control population, making it difficult to compare OGM results with RP‐PCR and LR‐GS. The size of the insertion in our samples was, on average, 600 bp, which is very close to the OGM limit of detection of 500 bp and could explain why some expansions were not recognized as an insertion by the *de novo* algorithm. The ability of OGM to detect repeat expansions has been demonstrated in over a dozen conditions caused by pathogenic repeat expansions, including neurodegenerative disorders such as Friedreich ataxia (*FXN*), spinocerebellar ataxia type 10 (*ATXN10*), spinocerebellar ataxia type 8 (*ATNX8*), Huntington disease (*HTT*), and CANVAS (*RFC1*) [24, 25, 26]. With the increasing number of repeat disorders detected by OGM, it is likely that the algorithms will be refined and will better detect these genetic variants in their future iterations. Unfortunately, because OGM does not sequence individual bases, but instead detects a specific fluorescently labeled pattern across the genome, it cannot delineate a pathogenic pure repeat motif from a non‐pathogenic impure one, a challenge also inherent to the PCR‐based methods [7]. One way to overcome this limitation is to use OGM as a first‐tier test to screen for ataxias in combination with next‐generation sequencing.

In conclusion, we have identified a pathogenic GAA expansion in intron 1 of *FGF14* in 65.2% of an unsolved adult‐onset ataxia cohort in the Care4Rare research program through a combination of molecular tools. Follow‐up studies should include a focused effort to increase diversity in the study cohort to determine the contribution of the pathogenic *FGF14* expansion in adult‐onset ataxia across Canada as a whole.

## Author Contributions

All authors contributed to the data generation and analysis in this study. A.C., G.F.D.G., and K.M.B. wrote the initial manuscript. All other authors reviewed and provided editorial input to the manuscript.

## Conflicts of Interest

The authors declare no conflicts of interest.

## Supporting information


**Data S1:** Supporting Information.
**Supplementary Table S1:** Haplotype comparison between FC patients from Quebec by Pellerin et al. 2023 and our FC participants. For each nucleotide, the position (pos), rsid, and values for our FC participant (haplotype_C4R) and the haplotype from Pellerin et al. 2023 (haplotype_Pellerin2023) are listed. Identical nucleotides between the two FC cohorts are highlighted in green and the ones that differ in red. The GAA repeat locus is highlighted in yellow.
**Supplementary Table S2:** Detailed clinical characterization and testing for all participants. All methods and results are listed for each participant, in addition to their ethnicity, family history, and phenotype.

## Data Availability

Data that support the findings of this study are available from the corresponding author upon reasonable request.
